# Transcriptome profiling of *Bacillus subtilis* OKB105 in response to rice seedlings

**DOI:** 10.1186/s12866-015-0353-4

**Published:** 2015-02-06

**Authors:** Shanshan Xie, Huijun Wu, Lina Chen, Haoyu Zang, Yongli Xie, Xuewen Gao

**Affiliations:** Department of Plant Pathology, College of Plant Protection, Nanjing Agricultural University, Key Laboratory of Integrated Management of Crop Diseases and Pests, Ministry of Education, Weigang No.1, Nanjing, 210095 People’s Republic of China

**Keywords:** *Bacillus subtilis*, *Oryza sativa*, Plant-microbe interactions, Transcriptomics, Microarray, Functional annotation

## Abstract

**Background:**

Plant growth-promoting rhizobacteria (PGPR) are soil beneficial microorganisms that colonize plant roots for nutritional purposes and accordingly benefit plants by increasing plant growth or reducing disease. However, the mechanisms and pathways involved in the interactions between PGPR and plants remain unclear. In order to better understand these complex plant-PGPR interactions, changes in the transcriptome of the typical PGPR *Bacillus subtilis* in response to rice seedlings were analyzed.

**Results:**

Microarray technology was used to study the global transcriptionl response of *B. subtilis* OKB105 to rice seedlings after an interaction period of 2 h. A total of 176 genes representing 3.8% of the *B. subtilis* strain OKB105 transcriptome showed significantly altered expression levels in response to rice seedlings. Among these, 52 were upregulated, the majority of which are involved in metabolism and transport of nutrients, and stress responses, including *araA*, *ywkA*, *yfls*, *mtlA*, *ydgG* et al. The 124 genes that were downregulated included *cheV*, *fliL*, *spmA* and *tua*, and these are involved in chemotaxis, motility, sporulation and teichuronic acid biosynthesis, respectively.

**Conclusions:**

We present a transcriptome analysis of the bacteria *Bacillus subtilis* OKB105 in response to rice seedings. Many of the 176 differentially expressed genes are likely to be involved in the interaction between Gram-positive bacteria and plants.

## Background

Plant growth-promoting rhizobacteria (PGPR) are soil microorganisms that colonize plant roots, obtaining nutritional benefits from the plant in exchange for stimulating plant growth and reducing plant disease. These benefical plant-microbe interactions are complex. Plants release chemicals such as malic acid that attract rhizobacteria, causing migration of microorganisms towards and along the roots [1-4]. After colonization, rhizobacteria consume carbohydrates and amino acids released by the plant. Simultaneously, PGPR produce substances affecting plant growth and development such as the plant hormones indole-3-acetic acid [5], cytokinins [6], and gibberellins [7]. PGPR also produce volatiles that promote plant growth [8], and they protect plants against soil-borne diseases by predation and parasitism of plant-hostile organisms, outcompeting plant pathogens for niches or specific substances such as nutrients or ferric iron. Furthermore, PGPR also produce antibiotics that work against plant pathogens, and can induce plant resistance directly [9,10]. Despite advances, it remains unclear exactly which mechanisms or pathways are involved in the interactions between PGPR and plants.

Recently, transcription microarray technology and comparative proteomic analysis have been applied to improve our understanding of plant-microbe interactions. To date, the focus has largely been on plant responses to benefical bacteria. A study in *Arabidopsis* showed that some putative auxin-regulated genes and nodulin-like genes were up-regulated, and some ethylene-responsive genes were down-regulated, following exposure to *Pseudomonas fluorescens* FPT9601-T5 [11]. In another study, rice proteins involved in plant growth and defence were induced after exposure to *Bacillus cereus* NMSL88 [12]. Proteins reported to be directly or indirectly involved in growth promotion were differentially expressed in rice following inoculation with *P. fluorescens* KH-1 [13]. Relatively fewer studies have focused on the transcriptional changes that occur in benefical bacteria when interacting with plants. A number of *P. aeruginosa* genes involved in metabolism, chemotaxis, and type III secretion were upregulated in response to sugar beet exudates [14]. Amino acids and aromatic compounds in root exudates were shown to induce *P. putida* to colonize the rhizosphere [15]. In another study, several groups of genes from *B. amyloliquefaciens* FZB42 were strongly induced by maize root exudates, most of which were involved in nutrient utilization, bacterial chemotaxis and motility, and non-ribosomal synthesis of antimicrobial peptides and polyketides [16]. These and other studies all investigated the effects of root exudates on PGPR, but studies on the effects of living plants on PGPR are needed if we are to understand the complex nature of plant-PGPR interactions.

*B. subtilis* OKB105 is a derivative of *B. subtilis* 168 that contains an *sfp* gene that encodes a phosphopantetheinyl transferase involved in surfactin production and that renders this strain with the ability to produce high levels of surfactin [17]. *B. subtilis* OKB105 has shown great potential as a growth-promoting and biocontrol agent. The microbe significantly enhanced plant height and fresh weight, lowered the severity of disease caused by tobacco mosaic virus, and exhibited nematicidal activity against *Aphelenchoides besseyi*, *Ditylenchus destructor*, *Bursaphelenchus xylophilus* and *Meloidogyne javanica* [18,19]. The mechanism by which OKB105 promotes plant growth and reduces disease are not fully understood. To address this question, we performed transcriptomics experiments to identify *B. subtilis* OKB105 genes that are differentially expressed in response to rice seedlings, and investigated their roles in plant-microbe interactions. To our knowledge, this is the first report on the transcriptomic responses of *Bacillus* spp. upon interaction with living plants.

## Results

### Effects of *B. subtilis* on rice growth

**Figure 1:**
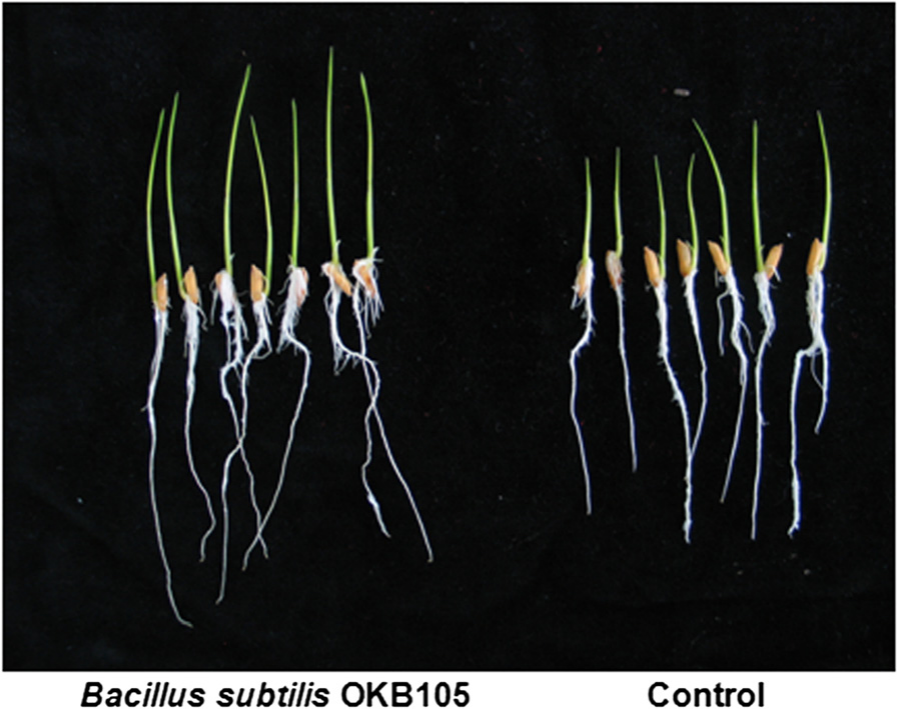
**Effect of** ***Bacillus subtilis*** **OKB105 on rice (cultivar 9311) growth.** Rice seeds (cultivar 9311) were soaked in *B. subtilis* OKB105 suspensions at cell densities of 10^6^ cfu ml^-1^ for 2 h, blotted dry and then placed in wet blotters and incubated in a growth chamber. The shoot and root lengths of rice seedlings were measured after 10 days.

The effects of *B. subtilis* OKB105 on rice growth was evaluated in this study. After surface sterilization, rice seeds were soaked in *B. subtilis* OKB105 cell suspensions, dried and incubated at 28°C. Shoot and root lengths of rice seedlings were measured after 10 days, and bacteria increased the shoot length by 25.2%, whereas discrepant analysis of root length showed no difference (Figure 1).

### Selection of appropriate interaction time

**Figure 2:**
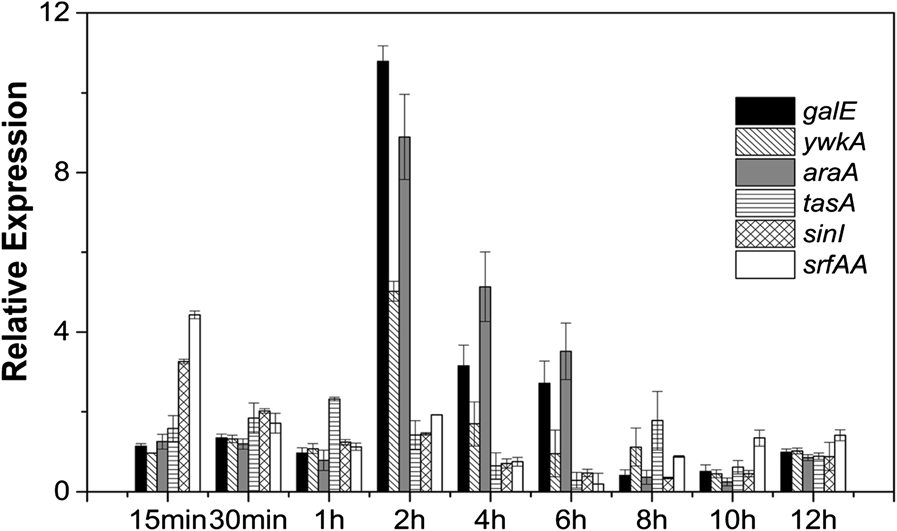
**Real**-**time PCR analysis of genes involved in biofilm formation and degradation of nutrients.** *B. subtilis* OKB105 cells were harvested at different times during interaction with rice seedlings (15 min, 30 min, 1 h, 2 h, 4 h, 6 h, 8 h, 10 h and 12 h) for extracting total RNA. The gene *galE* encoding UDP-glucose-4-epimerase, *ywkA* encoding malate dehydrogenase and *araA* encoding L-arabinose isomerase were identified, and are known to be involved in nutrient degradation. *TasA* encoding major a biofilm matrix component, *srfAA* encoding surfactin synthetase, and *sinI* encoding antagonist of SinR were also identified, and are involved in biofilm formation.

Plant-microbe interactions are a complex phenomenon and involve recognition, movement, colonization and production of metabolites from both organisms that influence the other. During the initiation phase of the interaction, plants release signals that attract bacteria via a chemotactic response [20], and that are consumed by the bacteria as an energy source. Plant root exudates affect many aspects of bacterial biochemistry and physiology including cell density, the types of bacteria present in the community, and migration towards and colonization of plant roots [21,22]. However, bacteria not in physical contact with rice seedlings can also have a great influence on plants. For example, volatiles produced by *Bacillus subtilis* promote growth and induce systemic resistance in *Arabidopsis* [8]. Communication without physical contact is therefore a type of interaction, and whole cell suspensions were collected and tested using realtime PCR analysis for this reason. In order to identify the onset of this early phase, expression levels of genes involved in biofilm formation and nutrient degradation were measured at different timepoints during the incubations. The chosen genes were as follows: *galE* encoding UDP-glucose-4-epimerase, *ywkA* encoding malate dehydrogenase, and *araA* encoding L-arabinose isomerase that are all involved in carbohydrate degradation; *tasA* encoding a major biofilm matrix component, *srfAA* encoding surfactin synthetase, and *sinI* encoding an antagonist of SinR that are all involved in biofilm formation. The results showed that the expression of *srfAA* and *sinI* were significantly altered after interacting with rice for only 15 min, part of cells may colonized on rice seedlings and biofilm involvement in response to rice seedlings. In contrast, genes involved in carbohydrate degradation did not undergo significant changes in expression until 2 h, indicating that most of the bacterial population had been exposed to the root exudates by this point (Figure 2). These results suggest that bacteria quickly become established in roots and begin utilizing plant carbohydrates after 2 h, therefore 2 h was chosen as an appropriate interaction time.

### Microarray analysis of *B. subtilis* OKB105 gene expression in response to rice

**Figure 3:**
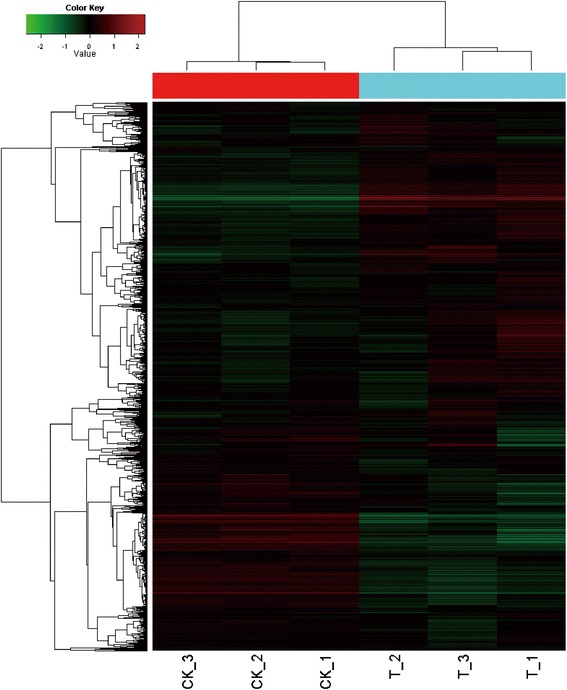
**Cluster analysis of microarray data.** CK, *B. subtilis* OKB105 without contact with rice seedlings; T, *B. subtilis* OKB105 after interaction with rice seedlings. The cluster analysis was performed using Cluster 3.0 software. Red and green indicate higher (>2.0) and lower (<0.5) ratios, respectively. Each treatment was repeated three times.

To investigate the molecular mechanisms involved in plant-microbe interactions, three independent experiments were carried out. To evaluate sample consistency, microarray data were analysed using cluster 3.0 software, and hierarchical analysis showed clearly defined groups for the three replicated experimental rice seedling samples and the three replicated control samples (Figure 3), indicating consistency.

**Figure 4:**
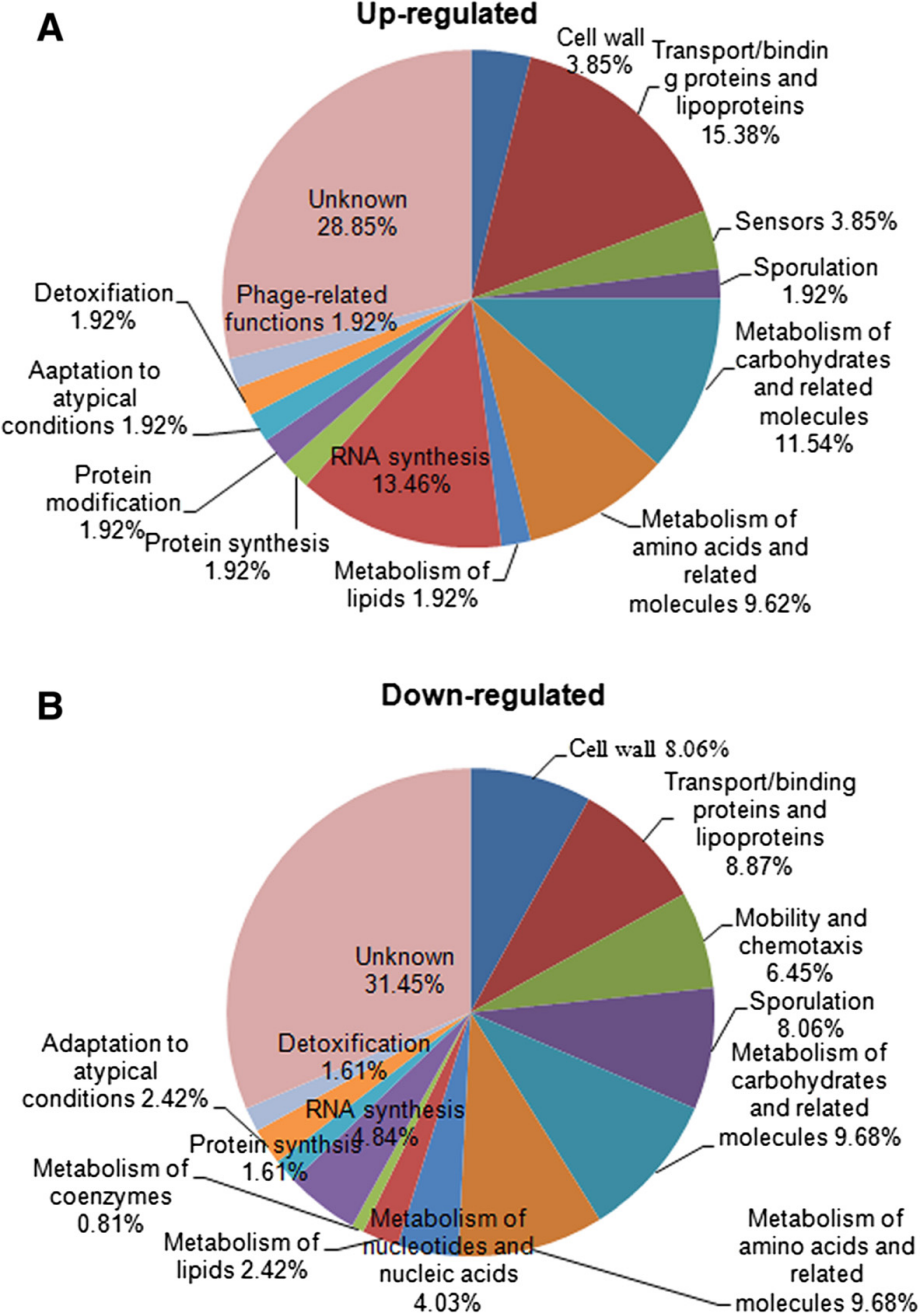
**Functional categories of** ***B. subtilis*** **OKB105 genes exhibiting altered transcription after interaction with rice seedlings.** 122 genes were of known function and classified accordingly, while 54 were of unknown function.

Differentially expressed genes were identified by the following selection criteria: (1) changes in gene expression occurred in the same direction in all three microarray analyses, (2) the average change in expression level was greater than 2-fold for up- and downregulated genes, (3) the q value was less than 0.05. When this criterion was applied, a total of 176 genes representing 3.8% of the transcriptome were significantly altered in response to rice seedlings. Among these differentially expressed genes, 52 were upregulated and 124 were downregulated (Table 1). In addition, a significant proportion (~30%) of the differentially expressed genes encoded proteins with putative functions or were described as ‘hypothetical proteins’ in the databases. The majority of the differentially expressed genes belonged to the following functional categories: Transport/binding proteins and lipoproteins (15.38% of upregulated genes, 8.87% of downregulated genes); RNA synthesis (13.46% of upregulated genes, 4.84% of downregulated genes); Metabolism of carbohydrates and related molecules (11.54% of upregulated genes, 9.68% of downregulated genes); Metabolism of amino acids and related molecules (9.62% of upregulated genes, 9.68% of downregulated genes); sporulation (8.06% of downregulated genes); Mobility and chemotaxis (6.45% of downregulated genes) (Figure 4). The majority were related to transport and metabolism, and this may be due to the importance of material and energy exchange between plant and *Bacillus*. Genes related to sporulation were downregulated. This may be because without nutrition provided by plant root exudates, nutrient deprivation and general stress triggers differentiation into dormant spores. In addition, it is viable cells rather than dormant spores that interact with rice seedlings.

**Table 1:** ***Bacillus subtilis*** **OKB105 genes differentially expressed in response to rice seedlings**

**Gene**	**Annotation**	**Fold**-**change ratio**	**q**-**value (%)**
Up-regulated genes			
Cell wall (3.85%)			
*yngB*	UTP-glucose-1-phosphate uridylytransferase	2.631	**0**
*dacA*	Penicillin-binding protein 5	2.2647	0
Transport/binding and lipoproteins (15.38%)			
*ydgH*	Putative drug exporter of the RND superfamily	16.1482	0
*mtlA*	PTS mannitol-specific enzyme II CB component	5.7777	0
*yhcA*	Multidrug resistance protein	5.2704	**0**
*ykoY*	Transporter	3.8449	0
*yxkD*	Efflux transporter	2.7977	0
*yflA*	Amino acid carrier protein	2.1064	0
*ydfM*	Cation efflux system	2.083	0
*yflS*	2-Oxoglutarate/malate translocator	2.0806	0
Sensors (signal transduction) (3.85%)			
*yclJ*	Two-component response regulator YclK	2.4004	0
*yclK*	Two-component sensor histidine kinase	2.3307	0
Sporulation (1.92%)			
*rapF*	Response regulator aspartate phosphatase	2.408	0
Metabolism of carbohydrates and related molecules (11.54%)			
*mtlD*	Mannitol-1-phosphate dehydrogenase	5.5707	0
*ywkA*	Malate dehydrogenase	4.12	0
*araA*	L-arabinose isomerase	2.7149	0
*galE*	UDP-glucose 4-epimerase	2.6384	0
*yngB*	UTP-glucose-1-phosphate uridylytransfarase	2.631	0
*mmgD*	Citrate synthase III	2.0082	
Metabolism of amino acids and related molecules (9.62%)			
*proB*	Glutamate-5-kinase	2.5409	0
*ald*	L-alanine dehydrogenase	2.3522	0
*speA*	Arginine decarboxylase	2.2631	0
*yrpC*	Glutamate racemase	2.0801	**0**
*hutH*	histidase	2.0154	0
Metabolism of lipids (1.92%)			
*yngG*	Hydroxymethylglutary-COA lyase	2.2458	**0**
RNA regulation (13.46%)			
*ydgG*	MarR family transcriptional regulator	13.515	0
*yhbI*	Transcriptional regulator (MarR family)	7.9016	0
*yhgD*	Transcriptional regulator (TerR/AcrR family)	7.5195	
*yhcB*	Trp repressor binding protein	2.5366	0
*fruR*	Transcriptional repressor of the fructose operon	2.4164	0
*yugG*	Lrp/Asnc family transcriptional regulator	2.2458	0
*yusO*	MarR family transcriptional regulator	2.1994	0
Protein synthesis (1.92%)			
*pheS*	Phenylalanyl-tRNA synthetase	2.0356	0
Protein modification (1.92%)			
*yxaL*	Serine/threonine protein kinase	2.3234	0
Adaptation to atypical conditions (1.92%)			
*rsbX*	Serine phosphatase	2.1821	0
Detoxification (1.92%)			
*ykoY*	Toxic anion resistance protein	3.4438	0
Phage-related functions (1.92%)			
*yhgE*	Phage infection protein	5.0703	0
Unknown (28.85%)			
*ydfK*	Putative integral inner membrane protein	3.8449	0
*yvpB*	Putative hydrolase	3.5212	0
*yneF*	Hypothetical protein	3.0863	0
*yrkO*	Putative integral inner membrane protein	2.6702	0
*yngA*	Putative conserved membrane protein	2.4775	0
*yhaJ*	Putative bacteriocin	2.4261	0
*yfkA*	Putative Fe-S oxidoreductase	2.4164	0
*yfhE*	Hypothetical protein	2.3804	0
*ykcB*	Putative integral inner membrane protein	2.2993	0
*ywkB*	Putative transporter	2.2629	0
*yaaT*	Hypothetical protein	2.2497	0
*ykaA*	Putative Pit accessory protein	2.0901	0
*ydhB*	Putative integral inner membrane protein	2.0405	0
*ykyB*	Hypothetical protein	2.0219	0
Down-regulated			
Cell wall (8.06%)			
*tuaD*	Biosynthesis of teichuronic acid	0.4536	0
*tuaA*	Biosynthesis of teichuronic acid	0.4382	0
*lytD*	N-Acetylglucosaminidase	0.4371	0
*tuaC*	Biosynthesis of teichuronic acid	0.435	
*tuaF*	Biosynthesis of teichuronic acid	0.3949	0
*tuaG*	Biosynthesis of teichuronic acid	0.3922	0
*tuaB*	Biosynthesis of teichuronic acid	0.3855	0
*pbpE*	Penicillin-binding protein 4	0.3219	0
*tuaH*	Biosynthesis of teichuronic acid	0.319	0
*tuaE*	Biosynthesis of teichuronic acid	0.2841	0
Transport/binding proteins and lipoproteins (8.87%)			
*opuD*	Glycine betaine transporter	0.491	0
*gabP*	γ-Aminobutyrate permease	0.4897	0
*ydhF*	Lipoproteins	0.4892	0
*iolF*	Inositol transport protein	0.4687	0
*yxlG*	ABC transporter permease	0.4536	0
*yxlF*	ABC transporter	0.3579	0
*glpF*	Glycerol uptake facilitor	0.3393	0
*yteP*	Transmembrane lipoprotein	0.3054	0
*ytcQ*	Lipoprotein	0.221	0
*ytcP*	ABC transporter	0.2021	0
*yybF*	Antibotic resistance protein	0.1847	0
Mobility and chemotaixs (6.45%)			
*fliL*	Flagellar protein required for flagellar formation		
*yvyG*	Flagellar protein	0.4886	0
*flgK*	Flagellar hook-associated protein 1 (HAP1)	0.4395	0
*hag*	Flagellin protein	0.3963	0
*fliK*	Flagellar hook-length control	0.3692	0
*flgL*	Flagellar hook-associated protein # (HAP3)	0.361	0
*cheV*	Modulation of cheA activity in response to attractants	0.3404	0
*fliJ*	Flagellar protein required for formation of basal body	0.2175	0
Sporulation (8.06%)			
*spmA*	Spore maturation protein	0.4981	0
*phrC*	Phosphatase regulator	0.4976	0
*cgeD*	Matyration of the outermost layer of the spore	0.4922	0
*usd*	Required for translation of spoIII D	0.4893	0
*rapG*	Response regulator aspartate phosphatase	0.4844	0
*tlp*	Small acid-soluble spore protein	0.4354	0
*phrE*	Phosphatase regulator	0.4216	0
*phrG*	Response regulator aspartate phosphatase	0.3595	0
*ywcE*	Protein required for proper spore morphogenesis and germination	0.1561	0
*csfB*	Forespore-specific protein	0.2353	0
Metabolism of carbohydrates and related molecules (9.68%)			
*abnA*	Arabinan-endo-1,5-α-L-arabinase	0.4833	0
*pdhB*	Pyruvate dehydrogenase	0.4766	0
*bglH*	β-Glucosidase	0.4239	0
*iolH*	Myo-inositol catabolism	0.4076	0
*iolG*	Myo-inositol catabolism	0.3885	0
*glpK*	Glycerol kinase	0.3478	0
*yteT*	Rhamnogalacturonyl dehydrogenase	0.299	0
*iolE*	Myo-inositol catabolism	0.2972	0
*iolD*	Myo-inositol catabolism	0.2712	0
*iolB*	Myo-inositol catabolism	0.2634	0
*iolC*	Myo-inositol catabolism	0.2609	0
*yteR*	Unsaturated rhamnogalacturonyl hydrolase	0.2221	0
Metabolism of amino acids and related molecules (9.68%)			
*argG*	Argininosuccinate synthase	0.49	0
*leuA*	2-Isopropylmalate synthase	0.4795	0
*yuxL*	Acylaminoacyl-peptidase	0.4718	0
*vpr*	Minor extracellular serine protease	0.4661	0
*leuD*	3-Isopropylmalate dehydratase	0.462	0
*ymfH*	Processing protease	0.4612	0
*leuB*	3-Inospropylmalate dehydratase	0.4476	0
*epr*	Minor extracellular serine protease	0.429	0
*argC*	N-Acetylornithine aminotransferase	0.394	0
*racX*	Amino acid racemase	0.3608	0
*ilvC*	Ketol-acid dehydratase	0.3524	0
*yaaO*	Lysine decarboxylase	0.2553	0
Metabolism of nucleotides and nucleic acids (4.03%)			
*purF*	Glutamine phosphoribosylpyrophosphate aminotransferase	0.4532	0
*purM*	Phosphoribosylglycinamidezole synthetase	0.4313	0
*purD*	Phosphoribosylglycinamide synthetase	0.4158	0
*purH*	Phosphoribosylglycinamide carboxy formyl formyltransferase	0.4008	0
*purN*	Phosphoribosylglycinamide formyltransferase	0.3243	0
Metabolism of lipids (2.42%)			
*yusK*	Acetyl-CoA C-acyltransferase	0.4973	0
*glpQ*	Glycerophosphoryl diester phosphodiesterase	0.4721	0
*yvaG*	3-Oxoacyl-acyl-carrier protein reductase	0.4424	0
Metabolism of coenzymes and prosthetic groups (0.81%)			
*folC*	Folyl-polyglutamate synthetase	0.3952	0
RNA synthesis (4.84%)			
*spo0A*	Two-component response regulator central for the initiation of sporulation	0.4924	0
*sigA*	RNA polymerase major sigma factor	0.4332	0
*abh*	Transcriptional regulator of transition state genes	0.4266	0
*yozG*	Transcriptional regulator	0.3154	0
*sigY*	RNA polymerase ECF-type sigma factor	0.2552	0
*ykoM*	Transcriptional regulator	0.2517	0
Protein synthesis (2.42%)			
*yxlE*	Negative regulator of sigma-Y antivity	0.2727	0
*yxlD*	Sigma-Y antisigma factor component	0.2629	0
*yxlC*	Sigma-Y antisigma factor	0.2276	0
Aminoacyl-tRNA synthetases (1.61%)			
*ileS*	Isoleucyl-tRNA synthetase	0.4435	0
*valS*	Valy-tRNA synthetase	0.3497	0
Detoxification (1.61%)			
*ybfO*	Erythromycin esterase	0.4784	0
*yndN*	Fosfomycin resistance protein	0.4711	0
Unknown (31.45%)			
*yisT*	Hypothetical protein	0.4827	0
*yukJ*	Hypothetical protein	0.4766	0
*ysdB*	Hypothetical protein	0.474	0
*ylxF*	Putative kinesin-like protein	0.473	0
*yrzF*	Putative serine/threonine-protein kinase	0.4723	0
*ywqH*	Hypothetical protein	0.4715	0
*ykpC*	Hypothetical protein	0.4711	0
*ypiB*	Hypothetical protein	0.4647	0
*yitR*	Hypothetical protein	0.4627	0
*yvfG*	Hypothetical protein	0.4602	0
*yhfM*	Hypothetical protein	0.4522	0
*yvaG*	Putative oxidoreductase	0.4424	0
*yqhO*	Hypothetical protein	0.4406	0
*ytzD*	Hypothetical protein	0.4349	0
*ywnF*	Hypothetical protein	0.4316	0
*yfmB*	Hypothetical protein	0.4297	0
*ypiF*	Hypothetical protein	0.4239	0
*yqhL*	Hypothetical protein	0.4205	0
*yjfB*	Hypothetical protein	0.4165	0
*yocB*	Hypothetical protein	0.4042	0
*yyaB*	Putative integral inner membrane protein	0.4036	0
*ytbQ*	Putative nucleoside-diphosphate-sugar epimerase	0.3851	0
*ybgB*	Hypothetical protein	0.3787	0
*yxbC*	Hypothetical protein	0.3598	0
*yrzL*	Hypothetical protein	0.3565	0
*yuiB*	Hypothetical protein	0.3557	0
*ykrP*	Putative integral inner membrane protein	0.3394	0
*yhzC*	Hypothetical protein	0.3291	0
*yyaO*	Hypothetical protein	0.2553	0
*yxbB*	Putative S-adenosylmethionine-dependent methyltransferase	0.2325	0
*yuiA*	Hypothetical protein	0.2091	0
*yxnB*	Hypothetical protein	0.1604	0

### Validation of microarray results by real-time PCR

**Figure 5:**
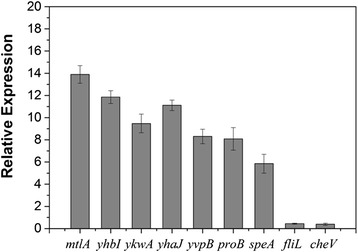
**Real**-**time PCR validation of differentially expressed genes.** Expression levels of randomly selected genes were measured using a 7500 Fast real-time PCR System. Statistically significant differences were determined using Fisher’s test (P ≤0.05).

Seven up-regulated and two down-regulated genes were chosen for evaluation by real-time PCR. All nine genes were confirmed as being differentially expressed in response to rice seedlings (Figure 5), which confirmed the reliability of the microarray data.

### Differentially expressed genes with known function

**Figure 6:**
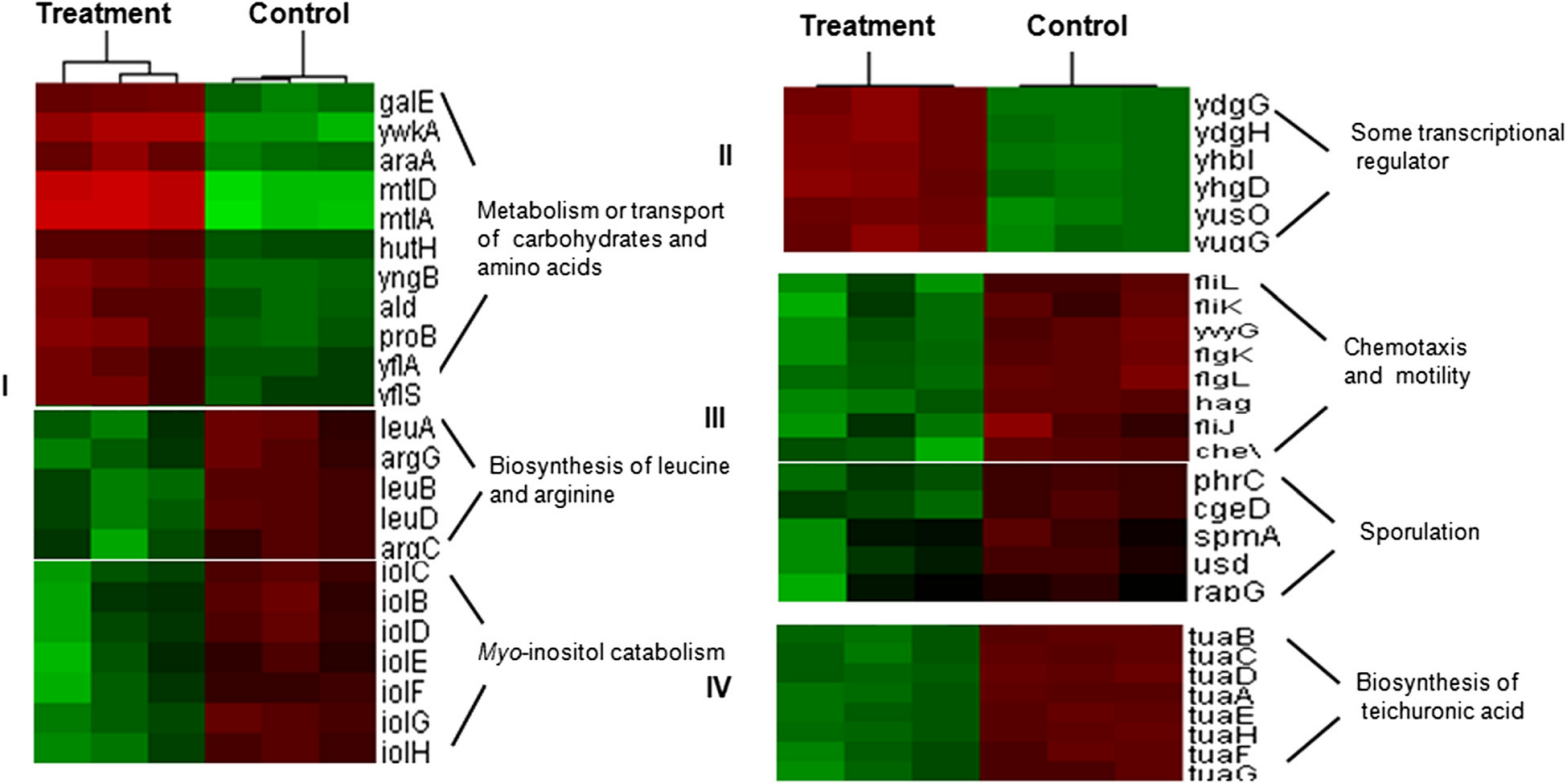
**A subset of** ***B. subtilis*** **OKB105 genes exhibiting altered expression in response to rice seedlings.** I, genes involved in metabolism or transport of carbohydrates and amino acids; II, genes associated with RNA synthesis including transcriptional regulators associated with stress responses; III, genes involved in chemotaxis, motility and sporulation; IV, genes associated with teichuronic acid biosynthesis. Cluster analysis was performed using cluster 3.0 software. Red and green indicate higher (>2.0) and lower (<0.5) ratios, respectively. Each treatment was repeated three times.

Among the 176 differentially expressed genes, 122 had known functions such as involvement in aspects of metabolism, transport, mobility, and chemotaxis. Of these, four groups of genes were particularly strongly affected by rice seedlings (Figure 6).

(1) 43 genes involved in metabolism or transport of carbohydrates or amino acids were significantly altered in response to rice seedlings. Of these, *galE* and *yngB* (galactose metabolism), *araA* (arabinose utilization) and *mtlD* (mannose metabolism) were upregulated in response to rice seedlings. Genes *proB*, *ald*, *hutH*, involved in proline, alanine, and histidine metabolism respectively, were also upregulated, while genes involved in leucine (*leuA*, *leuB*, *leuD*) and arginine (*argC*, *argG*) biosynthesis were downregulated. Genes encoding proteins involved in carbohydrate and amino acid transport such as *yfls*, *yflA*, and *mtlA* were also stimulated. This finding is perhaps not surprising because malate, glucose, arabinose, mannose, glucuronic acid, histidine, proline, leucine, alanine and arginine are all present in rice root exudates cultured in hydroponic conditions [23,24]. In this study, *ywkA* involved in malate metabolism, was upregulated. Malate has been reported to specifically attract *B. subtilis* in an isomer- and dose-dependent manner [22], suggesting that root exudates serve as energy sources and attractants in the interaction between roots and rhizobacteria. On the other hand, *B. subtilis* OKB105 cells not exposed to rice seedlings may be induced to sporulate due to a lack of energy that would otherwise be provided by the rice seedlings. Correspondingly, bacterial metabolism may remain low, and genes involved in the metabolism of carbohydrates or amino acids may be upregulated in response to rice seedlings.

Genes in the *B. subtilis* inositol operon (*iolB*, *iolC*, *iolD*, *iolE*, *iolF*, *iolG*, *iolH*), involved in *myo*-inositol catabolism, were downregulated. Previous reports suggested glucose is the main sugar found in rice root exudates, and DNA microarray results indicated that the *iol* operon was repressed by glucose through catabolite repression [23,25]. In summary, the presence of glucose inhibited the expression of genes involved in inositol metabolism.

(2) 13 genes associated with RNA synthesis including stress response transcriptional regulators were significantly upregulated. Among these, *ydgG* and *yhbI* showed the biggest changes (*ydgG*, 13.515-fold; *yhbI*, 7.9016-fold). These genes belong to the MarR transcriptional regulator family that has been reported to regulate expression of proteins conferring resistance to multiple antibiotics, organic components, detergents, and oxidative stress agents [26-28]. During growth, rice may produce compounds such as momilactone B and 5-resorcinol which are harmful to rhizobacteria [29,30]. Upregulation of stress-associated transcriptional regulators may assist rhizobacteria to adapt to environmental changes and confer a competitive advantage in the rhizosphere.

(3) The third group of genes associated with chemotaxis, motility and sporulation were downregulated. Amino acids and sugars in root exudates act as attractants that cause microorganisms to move towards roots [31]. We might expect chemotaxis and motility-associated genes such as *cheV*, *fliL*, and *flgK* to be upregulated, but these were downregulated in this study. This may be due to the different detection times employed. Upon initiation of the interaction process, bacteria recognize plant signals and move towards plant roots. Bacterial motility in the rhizosphere involves several processes such as chemotaxis, flagella-driven motility, swarming, and production of surfactants [32-35]. Expression of *srfAA* and *sinI* were significantly altered after interacting with rice for only 15 min (Figure 2), indicating that the bacteria may well have finished migrating towards the roots by 2 h. In order to conserve energy, expression of genes associated with chemotaxis and motility could remain at a low level. In addition, the rapid surface motility of bacteria may be independent of flagella [36], which may also explain the downregulation of these genes.

Sporulation in *B. subtilis* can be induced by starvation of carbon, nitrogen and phosphorus. In this study, five genes (*spmA*, *phrC*, *cgeD*, *usd*, *rapG*) involved in sporulation were downregulated after interaction with rice. This may be explained by root exudates supplying the energy required for the dynamic *B. subtilis* cells. Alternatively, *B. subtilis* can lie dormant as if energy is in short supply or when encountering a hostile environment. During plant-microbe interactions both plants and PGPR receive mutual benefits, and it is viable microbial cells rather than dormant spores that interact with rice seedlings. This may explain why genes related to sporulation were down-regulated during the detection time in this study.

(4) The fourth group of genes exhibiting altered expression levels upon interaction with rice seedlings were associated with teichuronic acid biosynthesis. Anionic polymers make up 35-60% of the entire dry weight of the vegetative cell wall in *B. subtilis*, of which teichoic and teichuronic acids are the main types. When phosphate is sufficient, teichoic acids are present, whereas teichuronic acids predominate under phosphate-limiting conditions [37-39]. In this study, the *tua* operon (*tuaA*, *tuaB*, *tuaC*, *tuaD*, *tuaE*, *tuaF*, *tuaG*, *tuaH*) involved in teichuronic acid biosynthesis was repressed in response to rice seedlings, indicating that phosphate was sufficient and non-limiting in the rice-*B. subtilis* OKB105 interaction.

## Discussion

In the present study, a global analysis of transcription in *B. subtilis* OKB105 in response to rice seedlings was performed using microarray experiments. A total of 43 genes associated with metabolism or transport of carbohydrates and amino acids exhibited differential expression. Genes involved in metabolism or transport of carbohydrates and amino acids were upregulated, while genes associated with amino acid biosynthesis were downregulated. Genes involved in inositol metabolism (*iolB*, *iolC*, *iolD*, *iolE*, *iolG*, *iolH*) were also downregulated due to suppression by glucose [40]. Nearly a quarter of the genes exhibiting altered expression were involved in transport or utilization of nutrients, suggesting rhizobacteria use carbohydrates and amino acids released by plants as energy sources.

Transcriptional regulators associated with stress responses were also affected. Rice seedlings not only produce nutrients but also release harmful compounds such as momilactone B and 5-resorcinol [29,30]. In response to these harmful substances, several genes belonging to the MarR family of transcriptional regulators were upregulated. MarR family proteins have been reported to regulate the expression of proteins conferring resistance to multiple antibiotics, organic components, detergents, and oxidative stress agents [26-28]. This observation may reflect the adaptability of *B. subtilis*, which is important among the highly competitive microbial communities vying to reside in the rhizosphere.

Many *Bacillus* species have been reported to stimulate plant growth under different conditions [41,42]. The beneficial effects conferred by *Bacillus* species on plants may operate directly via enhanced provision of nutrients, phytohormones or volatiles, or indirectly through production of antibiotics and induction of plant resistance mechanisms (ISR) [8]. In the present study, *B. subtilis* OKB105 suspension increased shoot length by 25.2%, while genes related to plant growth were not up or downregulated. This phenomenon may be explained by at least three reasons: (1) plant-microbe interactions are highly complex. Within the time frame of the experiments conducted, *B. subtilis* began to utilize nutrients released by the plant and started to adapt to the changing environment, but the time for production of plant growth promoting substances may have been insufficient, (2) not all bacterial cells in contact with plants necessarily establish a productive interaction, therefore the effects may be diluted below detection levels as the cell populations are averaged, (3) the functions of some differentially expressed genes remain unknown, and this increases the difficulty of studying plant-microbe interactions. Determination of the molecular mechanisms involved in plant-rhizobacteria interactions requires much further study.

## Conclusion

Global analysis of transcription in *B. subtilis* OKB105 in response to rice seedlings was performed using microarray experiments. A total of 176 genes representing 3.8% of the *B. subtilis* strain OKB105 transcriptome showed significantly altered expression levels in response to rice seedlings. Differentially expressed genes were mainly involved in metabolism and transport of nutrients, stress responses, chemotaxis, motility, sporulation and teichuronic acid biosynthesis. The results had indicated that *B. subtilis* OKB105 could utilize carbohydrates and amino acids released by rice as energy sources, and then OKB105 migrates towards and establishes a relationship with rice. During the interaction process, OKB105 may enhance self-adaptability and cell viability in rhizosphere by inducing the expression of some transcriptional regulators and repressing sporulation-related genes expression, respectively. However, potential genes related to plant growth were not detected. More studies are needed to illustrate the nature of the complex plant-microbe interactions.

## Methods

### Preparation of *B. subtilis* OKB105 suspension

*B. subtilis* OKB105 was grown in LB at 37°C for 12 h. Cells were harvested by centrifugation at 8000 rpm for 15 min at 4°C, resuspended in distilled water and adjusted to a final concentration of 10^6^ cfu ml^-1^.

### Effects of *B. subtilis* on rice growth

The growth-promoting activity of *B. subtilis* OKB105 on rice was tested according to the standard roll towel method [14]. Rice seeds (cultivar 9311) were surface sterilized with 70% (v/v) ethanol for 1 min, disinfected with 5% (w/v) sodium hypochlorite for 15 min, and washed three times with sterile distilled water. After surface sterilization, rice seeds were soaked in bacterial suspensions for 2 h, blotted dry, placed in wet blotters and incubated in a growth chamber at 28°C for 10 days. Seeds soaked in sterile water were used as the control. The shoot and root lengths of rice seedlings were measured after 10 days. Each treatment included 30 seedlings and was repeated five times, and all experiments were repeated three times.

### *B. subtilis*-rice interactions

Surface-sterilized rice seeds were germinated in Petri dishes at 28°C for 3 days. Germinated seeds were transplanted into 10 individual sterile vessels, and each vessel containing 30 rice seedings was incubated at 28°C. After 7 days of growth, 50 ml 10^6^ cfu/ml *B. subtilis* OKB105 suspension was added. To determine the most suitable interaction time for subsequent transcription microarray analysis, rice seedlings were removed at 15 min, 30 min, and 1, 2, 4, 6, 8, 10 and 12 h. OKB105 cells not in physical contact with rice seedlings and cells washed from roots were collected by centrifugation at 4000 rpm for 30 min at 4°C and used for RNA extraction. *B. subtilis* OKB105 suspensions not interacting with rice were used as a control. Expression of genes involved in biofilm formation and nutrient degradation was detected using real-time PCR. The experiment was repeated three times.

### Total RNA extraction and microarray analysis

50 ml 10^6^ cfu/ml *B. subtilis* OKB105 suspensions incubated with 30 rice seedlings for 2 h were harvested for microarray analysis, and OKB105 cells not interacting with rice were used as a control. Total RNA was extracted using the Bacterial RNA Kit (Omega Bio-Tek, Norcross, GA, USA) according to the manufacturer’s instructions. Random priming cDNA synthesis, cDNA fragmentation and terminal labeling with biotinylated GeneChip DNA labeling reagent, and hybridization to the Affymetrix *Bacillus subtilis* Genome Array GeneChip were carried out by CapitalBio (CapitalBio Corporation, Beijing, China). The *Bacillus subtilis* genome array was custom designed by Affymetrix (Santa Clara, Calif.) using the published DNA sequence (GenBank accession no. NC_000964) [43]. The array contained probe sets to interrogate approximately 4,350 ORFs and 600 intergenic regions with an additional 45 control probe sets, and detects the antisense strand of the *B. subtilis* transcript. This design was completed as a custom design for Genencor International, Inc. in June 1998 and made available broadly in 2002. EST data used in the experimental design was from the *Bacillus subtilis* Genome Sequencing Project of the Institut Pasteur 10.1, December 1997. Each treatment was repeated three times.

Microarray data were collected and analyzed using the Affymetrix GeneChip Command Console software. And then further analyzed using hierarchical clustering with average linkage. Finally, tree visualization was performed with Java Treeview (Stanford University School of Medicine, Stanford, CA, USA). Transcripts were designated as significantly differentially expressed when they exhibited at least a 2-fold change in expression level and a q value of less than 0.05.

### Real-time PCR

First-strand cDNA was synthesized using reverse transcriptase (TaKaRa Bio Inc, Dalian, China) with random hexamer primers. Real-time PCR was performed using SYBR Premix Ex Taq polymerase (TaKaRa Bio Inc, Dalian, China) with an ABI 7500 Fast Real-time PCR System (Applied Biosystems, Foster City, CA, USA). 16S rRNA was used to normalize RNA levels. Expression levels of *galE*, *yywkA*, *araA*, *sinI*, *tasA* and *srfAA* were measured at different timepoints during the plant-microbe interactions. Microarray results were validated by measuring the expression levels of some randomly chosen differentially expressed genes.

### Microarray data accesion number

Microarray data have been deposited in the GEO database (http://www.ncbi.nlm.nih.gov/geo/) under accssion number GSE62421.

## Data analysis

Statistical analysis was carried out by Fisher’s least-significant difference test (P ≤ 0.05) using SPSS software.
